# Consequences of chronic bacterial infection in *Drosophila melanogaster*

**DOI:** 10.1371/journal.pone.0224440

**Published:** 2019-10-24

**Authors:** Moria Cairns Chambers, Eliana Jacobson, Sarah Khalil, Brian P. Lazzaro

**Affiliations:** 1 Department of Entomology, Cornell University, Ithaca, New York, United States of America; 2 Department of Biology, Bucknell University, Lewisburg, PA, United States of America; 3 Cornell Institute of Host-Microbe Interactions and Disease, Cornell University, Ithaca, New York, United States of America; Uppsala University, SWEDEN

## Abstract

Even when successfully surviving an infection, a host often fails to eliminate a pathogen completely and may sustain substantial pathogen burden for the remainder of its life. Using systemic bacterial infection in *Drosophila melanogaster*, we characterize chronic infection by three bacterial species from different genera - *Providencia rettgeri*, *Serratia marcescens*, and *Enterococcus faecalis–*following inoculation with a range of doses. To assess the consequences of these chronic infections, we determined the expression of antimicrobial peptide genes, survival of secondary infection, and starvation resistance after one week of infection. While higher infectious doses unsurprisingly lead to higher risk of death, they also result in higher chronic bacterial loads among the survivors for all three infections. All three chronic infections caused significantly elevated expression of antimicrobial peptide genes at one week post-infection and provided generalized protection again secondary bacterial infection. Only *P*. *rettgeri* infection significantly influenced resistance to starvation, with persistently infected flies dying more quickly under starvation conditions relative to controls. These results suggest that there is potentially a generalized mechanism of protection against secondary infection, but that other impacts on host physiology may depend on the specific pathogen. We propose that chronic infections in *D*. *melanogaster* could be a valuable tool for studying tolerance of infection, including impacts on host physiology and behavior.

## Introduction

Animal physiology is profoundly impacted by constant association with microbes. Commensal microbes that live on epithelial surfaces, in the gut, and in other body compartments have recently come to prominent attention. Another source of microbial association comes from pathogenic infections that remain in the host even after symptoms resolve. We and others have observed that many bacterial infections in *Drosophila melanogaster* are never eradicated; they are only controlled into chronic persistence [1–5]. Previous research has focused on the processes leading to chronic infection and the consequent immune stimulation [1–3,5]. However, aside from a small set of studies evaluating the reproductive output of females [1,4], there has been less effort focused on the longer term consequences of chronic infection.

Our work expands our understanding of chronic infection using three bacterial pathogens—*Providencia rettgeri*, *Serratia marcescens*, and *Enterococcus faecalis–*which are frequently employed in experimental infection of *D*. *melanogaster*. Most mortality from these infections occurs within three days post-infection [1,2,5–9]. Individuals that survive sustain stable bacterial loads for the remainder of their life with minimal impact on life-span in the laboratory [2]. Chronic pathogen burdens for these bacterial species can be as high as ten thousand colony forming units (CFU) per fly [1,2,5,9,10].

Sustaining an infection for the remainder of life may have immunological and physiological consequences for the host. The presence of bacteria and bacterially-derived peptidoglycan stimulates the *D*. *melanogaster* immune system [11–18]. Induced expression of antimicrobial peptide genes is positively correlated with bacterial load during lethal infections in *D*. *melanogaster*, the red flour beetle (*Tribolium castaneum)* and the buff-tailed bumblebee (*Bombus terrestris*) [19–21]. In the mealworm beetle (*Tenebrio molitor*), sustained bacterial infection is associated with continued expression of antimicrobial peptide genes [22,23]. These observations led us to hypothesize that chronically infected *D*. *melanogaster* may be in a state of chronic immune stimulation, which may be protective against future infection. For example, previous studies have shown that immune stimulation after clearing a live bacterial infection, or after injection of heat-killed bacteria, provides some protection against future infections in *D*. *melanogaster* [24,25]. Similar effects have been seen in other insect species [26,27]. However, on the other hand, compounding combinatorial infection could overwhelm the fly and cause increased probability of death. The strength of protection in *D*. *melanogaster* varies depending on the bacterial species of both the initial inoculation and secondary challenge [24,25], and lingering live bacteria during chronic infection might continue to damage the host.

In this work, we determine the consequences of chronic infection with three extracellular opportunistic pathogens: *Providencia rettgeri*, *Serratia marcescens*, and *Enterococcus faecalis*. These data provide insight into the physiological consequences of chronically sustained extracellular bacterial infection in *D*. *melanogaster*, establishing the foundation for future studies on tolerance of infection in *Drosophila* and other insects.

## Materials and methods

### *D*. *melanogaster* strains and husbandry

Unless specified otherwise, experiments were done using wild-type Canton-S (CS, Bloomington stock # 1). Immune reporter flies that express GFP under promoters from the antibacterial peptide genes *attacin A*, *diptericin A*, and *drosomycin* [28] were used for visual readout of the immune response. The flies were reared as described previously in Chambers et al. [2]. For rearing each line, approximately fifteen males and fifteen females were placed in plastic bottles with 50mL of a glucose-cornmeal-yeast medium (50g/L yeast, 60g/L cornmeal, 40g/L glucose, 7g/L *Drosophila* Agar, 12mL of Acid Mix (41.5mL Phosphoric Acid + 418mL Propionic Acid + 540.5mL distilled water) and 26.5mL/L methylparaben (Moldex)) and allowed to lay eggs for two days. Adults were cleared and offspring were given ten days to develop. Eclosed adults were transferred to a fresh bottle, kept overnight to allow mating, and sorted on the subsequent day into vials of twenty males. Flies were aged for four additional days before injection, such that all experiments were performed with flies aged 5–7 day post-eclosion. Experiments were performed with male flies, which were flipped to new vials every seven days to keep flies on relatively fresh food. All flies were kept at 25°C in an incubator with a 12:12 hour light-dark cycle.

### Bacterial strains and infection conditions

Three bacterial species were used: *Providencia rettgeri* (strain Dmel, isolated as an infection of field-caught *D*. *melanogaster* [29]), *Serratia marcescens* (strain BPL, an attenuated strain derived from the Type strain ATCC 13880 [30]) and *Enterococcus faecalis* (isolated as an infection of field-caught *D*. *melanogaster* [29]).

Bacterial stocks were maintained and infection procedures were performed as described previously [2,31]. Bacterial stocks were stored at -80°C in Luria Bertani (LB) broth containing 15% glycerol. Bacteria were first streaked on an LB agar plate from the stock, grown overnight at 37°C, and subsequently stored at 4°C for up to a month. For infection, liquid bacterial cultures were inoculated from a single bacterial colony from the plate and grown overnight for 16–20 hours. *P*. *rettgeri* and *S*. *marcescens* were grown in 2ml LB broth at 37°C with shaking in an orbital shaker at 120 rotations per minute. *E*. *faecalis*, which is microaerophilic, was grown in 2mL BHI broth at 37°C standing. The overnight cultures were then diluted in Phosphate Buffer Saline (PBS, pH 7.4) to a desired optical density (Absorbance_600_). A volume of 23nL of bacterial suspension was injected into each fly using a Nanoject II (Drummond, www.drummondsci.com) as described in Khalil et al. [31]. To initiate chronic infection, injections were done in the abdomen to minimize mortality [2] allowing us to efficiently focus on later stages of infection. For experiments where we wanted to initiate lethal secondary infection, we injected bacteria into the thorax because this generates a higher bacterial burden and more lethal infection [2]. Bacterial suspensions were generally injected at an optical density OD (A_600_) of 0.1, which corresponds to a dose of about 2,000–4,500 Colony Forming Units (CFU; viable bacteria) per fly for *P*. *rettgeri*, 2,000–6,000 CFU for *S*. *marcescens* and 2,000–4,000 CFU for *E*. *faecalis*. In the experiment explicitly testing infectious dose, other densities were used as needed. Sterile PBS was injected as a control. Flies were anesthetized with light CO_2_ for less than five minutes during the infection procedure. All controls were exposed to CO_2_ for the same amount of time and no CO_2_-induced mortality was observed. Infected flies were kept in groups with a maximum of 20 per vial (typically 16–20 flies) at 25°C in an incubator with a 12:12 hour light-dark cycle.

### Infectious dose survival experiments

For each experimental treatment, flies were injected in the abdomen with bacterial suspensions ranging from OD of 0.001 to 5.0 or with PBS as a control. Flies were housed in groups, with a maximum of 20 flies per vial (typically 18–20). Experimental blocks contained 2–3 vials of flies per experimental treatment, and there were three experimental blocks (independently performed experiments with a new bacterial culture and set of flies) completed for each bacterial species. After inoculation, death was recorded daily for two weeks, with dead flies left in the vials. After one week, surviving flies were transferred to fresh vials, so the number of flies per vial was subsequently more variable as it was dependent on mortality that occurred prior to transfer. Survival curves were plotted as Kaplan-Meier plots using Graphpad Prism 7.0. Statistical significance was determined using Cox Proportional Hazards Mixed Effects Model (*coxme*) with the package “Coxme” in R (http://www.r-project.org/). Fixed effects included were experimental block (B) and the infectious dose (D), and the interaction between B and D. Fixed effects are incorporated in sequence and factors are listed in order of inclusion in result tables. To account for the experimental structure, vial (V) nested inside of both B and D was included as a random effect. Infectious dose was estimated by using the optical density of the injected bacterial suspensions. Our models fulfill the assumption of Cox Proportional Hazards in that the proportional hazards do not cross over-time.

Model A: coxme(status,time) = B + D + (B x D) +(1|B/D/V)

### Bacterial load

For each experimental block, approximately 120 flies were injected in the abdomen with bacterial suspensions at each optical density (0.001, 0.01, 0.1, and 0.5) or injected with PBS as control. Flies were housed in groups, with a maximum of 20 flies per vial (typically 18–20) at 25°C. Pathogen burden at various time points post-infection was determined as the number of colony forming units (CFU) recovered from homogenized flies as described previously by our research group [2,31]. *P*. *rettgeri* and *S*. *marcescens* bacterial loads were determined using a WASP II autoplate spiral plater (Microbiology International). In these experiments, six or more flies from the same vial were individually homogenized in 250uL of PBS at the relevant time-point. For zero hour time-points, flies were placed on ice within two minutes of injection and homogenized within thirty minutes. Infection conditions expected to yield bacterial loads greater than 10^4^ CFU/fly were diluted ten-fold. The spiral plater lays 50uL of homogenate in a continuously decreasing concentric spiral on an LB plate. Plates were grown overnight at 37°C and then counted using the EZ-Count Automated Colony Counter (Microbiology International), which calculates the number of CFU per fly based on the number of colonies and their position along the spiral, allowing for effective resolution of three orders of magnitude on a single plate. *E*. *faecalis* load was determined using spot-plating due to high variability in load among individuals, making prediction of the appropriate dilution for spiral plating difficult. In order to conserve plates, we moved to spot plating, which can resolve bacterial load over six orders of magnitude on a single plate but is more technically difficult. In these experiments, 8–16 individual flies from the same vial were collected at each time point. Each individual fly was homogenized in 100uL, then the homogenate was diluted serially 1:10, 10uL of each dilution spotted onto LB agar and grown overnight at 37°C. Spots containing 30–300 colonies were counted and used to calculate CFU per fly. For all experiments, flies injected with sterile PBS were homogenized and plated to test for contamination and experiments where growth was detected in controls were discarded. Three experimental blocks were completed for both *P*. *rettgeri* and *E*. *faecalis*, while two experimental blocks were completed for *S*. *marcescens* because one block was discarded due to contamination found in sterile PBS injected controls. Analysis of variance (ANOVA) was used to determine the impact of infectious dose on bacterial loads one week and two weeks post-infection using packages “lme4” and “nlme” in R (http://www.r-project.org/). Factors included were log_10_ average infectious dose (D) and block (B). Infectious dose was estimated by using the average bacterial load at zero hours post-infection within each experimental block. Each factor is incorporated in sequence and factors are listed in order of inclusion in result tables. All models reported fulfilled assumptions of normality as determined by the Shapiro Test.

Model B: log_10_(persistent bacterial load) = B + D + (B x D)

### Quantitative RT-PCR

For each experimental block, groups of sixty flies were inoculated in the abdomen with either *P*. *rettgeri*, *E*. *faecalis* or *S*. *marcescens* and sixty flies were injected with sterile PBS in the abdomen. Following injection, the flies were housed in vials in groups of twenty and left at 25°C for seven days, during which time mortality remained under twenty percent. Surviving flies were sorted into groups of fifteen, homogenized in TRIzol (Life Technologies) and stored at -80°C until further processing. Three experimental blocks were completed, each with three homogenates for each condition. Samples were processed as described previously by our research group [2]. RNA was isolated using a TRIzol extraction, RNA samples were treated with DNase (Promega), and cDNA was generated using MLV-RT (Promega) and then diluted 1:20 in water. Quantitative PCR was performed using the SSO Advanced SYBR Green Kit (Bio-Rad) following the manufacturer’s directions, scaled down for 15μL reactions. For consistency across studies, we used the same qPCR primers as previous studies [2,31,32]. To allow calculation of primer efficiency, a standard curve generated by ten-fold serial dilutions of pooled cDNA was run on each plate. Primer sequences are reported in Table 1. For each cDNA sample, three technical replicates were performed for each primer set and the average threshold cycle (Ct) was used for further analysis. Relative expression values for each sample were calculating using the Pfaffl method, which is similar to the commonly used 2^-ΔΔCt^ method except that it uses experimentally determined amplification efficiencies instead of assuming perfect efficiency for every set of primers [33]. In these analyses, expression levels of the gene of interest are normalized to a reference gene (*rp49*). The threshold cycles for the reference gene were all within one Ct, indicating that there was consistent isolation and synthesis of cDNA across all samples. The output of the Pfaffl method for each sample was then used as the response variable in an ANOVA performed using the “aov” command in R. Factors included were experimental block (B), infection treatment (T: Sterile PBS, *P*. *rettgeri*, *S*. *marcescens*, or *E*. *faecalis*) and the interaction between them.

**Table 1:** Primers for qRT-PCR.

Gene	Forward		Reverse
*Rp49*	5’ AGGCCCAAGATCGTGAAGAA 3’		5’ GACGCACTCTGTTGTCGATACC 3’
*diptericin A*	5’ GCGGCGATGGTTTTGG 3’		5’ CGCTGGTCCACACCTTCTG 3’
*defensin*	5’ GAGGATCATGTCCTGGTGCAT 3’		5’ TCGCTTCTGGCGGCTATG 3’
*attacin A*	5’ CGTTTGGATCTGACCAAGG 3’		5’ AAAGTTCCGCCAGGTGTGAC 3’
*drosomycin*	5’ CTGCCTGTCCGGAAGATACAA 3’		5’ TCCCTCCTCCTTGCACACA 3’
*metchnikowin*	5’ AACTTAATCTTGGAGCGATTTTTCTG 3’		5’ ACGGCCTCGTATCGAAAATG 3’

Model C: Log_2_(relative expression) = B + T+ (B x T)

Each factor is incorporated in sequence and factors are listed in order of inclusion in result tables. Pairwise comparisons between individual conditions were determined by Tukey’s multiple comparison tests using the “TukeyHSD” command in R. Expression levels for all five AMP genes were assessed using the same fly homogenates, so to control for multiple testing we used a Bonferroni correction, which divides the threshold p-value by the number of tests, lowering our significance threshold to p-values < 0.01 instead of 0.05. Since this correction assumes that all tests are independent, it is conservative in the current context where the tested genes are at least partially coordinately regulated.

### Microscopy of GFP reporter flies

Ten flies of each reporter line were injected with 23nL of sterile media or bacterial suspension in the abdomen, with a goal of recovering at least six surviving flies for imaging (anticipating about 10–20% mortality). One week after injection, surviving GFP-reporter flies were anaesthetized on ice for 30 min. Flies were then imaged in groups of six using both brightfield and epifluorescent illumination with a Leica M165 FC microscope fitted with a Leica DFC 450 camera. Images were captured with Leica Application Suite V4.0. Images for all settings were captured in a single sitting using identical settings on the microscope.

### Secondary infection experiments

For each experiment, flies were first injected in the abdomen with bacterial suspensions (OD 0.1) to initiate chronic infection or injected with sterile PBS as control. The injections were done in the abdomen to minimize mortality [2] allowing us to efficiently focus on later stages of infection. Flies were housed in groups with a maximum of 20 flies per vial (typically 18–20). Infected flies were kept at 25°C in an incubator with a 12:12 hour light-dark cycle for one week to allow chronic infection to develop, during which time mortality remained under twenty percent. To initiate lethal secondary infection, surviving flies were then injected with either bacterial suspensions (OD 0.1) or sterile PBS as a control. Secondary injections were done in the thorax, which generates a more lethal infection, although delivery of sterile PBS through this route does not cause significant mortality [2]. Experimental blocks contained 2–3 vials of 20 flies per experimental treatment. All chronic infection-secondary infection pairings were repeated in three independent blocks except for the *P*. *rettgeri*-*P*. *rettgeri*, *P*. *rettgeri*-*S*. *marcescens* and *E*. *faecalis*-*E*. *faecalis* experiments, which were repeated in four independent blocks. After secondary infection, death was recorded daily for one week. Survival curves are plotted as Kaplan-Meier plots using the “survival” package in R. As there was minimal mortality of control flies injected with sterile PBS during the secondary injection, statistical analysis was performed on only flies that received live bacterial infection during the secondary bacterial injection. The impact of chronic infection on survival after secondary infection was determined using Cox Proportional Hazards Mixed Effects Model (*coxme*) with the package “coxme” in R (http://www.r-project.org/). Each factor is incorporated in sequence and factors are listed in order of inclusion in result tables. Fixed effects included were experimental block (B), infection treatment (T: media control or bacterial infection) and the interaction between B and T. To account for experimental structure, Vial (V) nested inside both B and T was included as a random effect. Our models fulfill the assumption of Cox Proportional Hazards in that the proportional hazards do not cross over-time.

Model D: coxme(status,time) = B + T + (B x T) + (1|B/T/V)

### Starvation assay

For each experiment, flies were first injected in the abdomen with bacterial suspensions (OD 0.1) to initiate chronic infection or injected with sterile PBS as control. The flies were housed in groups of 20 flies per vial, during which time mortality stayed under twenty percent. One week after injection, surviving flies (16–20 per vial) were flipped to vials containing 1% agarose and monitored for survival approximately every hour for 35 hours. These vials were kept in storage containers surrounded by damp paper towels to keep the environment humid and ensure that flies did not die from desiccation. Three experimental blocks were completed for both *S*. *marcescens* and *E*. *faecalis*, while six experimental blocks were completed for *P*. *rettgeri*. Survival curves were plotted as Kaplan-Meier plots using Graphpad Prism 7.0 and statistical significance was determined using Cox Proportional Hazards Mixed Effects models (*coxme–*Model D, described above) with the package “coxme” in R (http://www.r-project.org/). Our models fulfill the assumption of Cox Proportional Hazards in that the proportional hazards do not cross over time.

## Results

### Infectious dose impacts both survival and the persistent bacterial load

It is a common observation that larger inoculation doses lead to a greater risk of host death [5,21,24,34–37]. Perhaps less intuitively, increasing the initial inoculum of *P*. *rettgeri* also increases the chronic bacterial load sustained by survivors [5]. To test whether the relationship between infectious dose and chronic burden is general, we determined the bacterial load in flies that survive infection with *P*. *rettgeri*, *S*. *marcescens* and *E*. *faecalis* across a 5000-fold range of initial infectious doses over two weeks post-infection.

**Fig 1:**
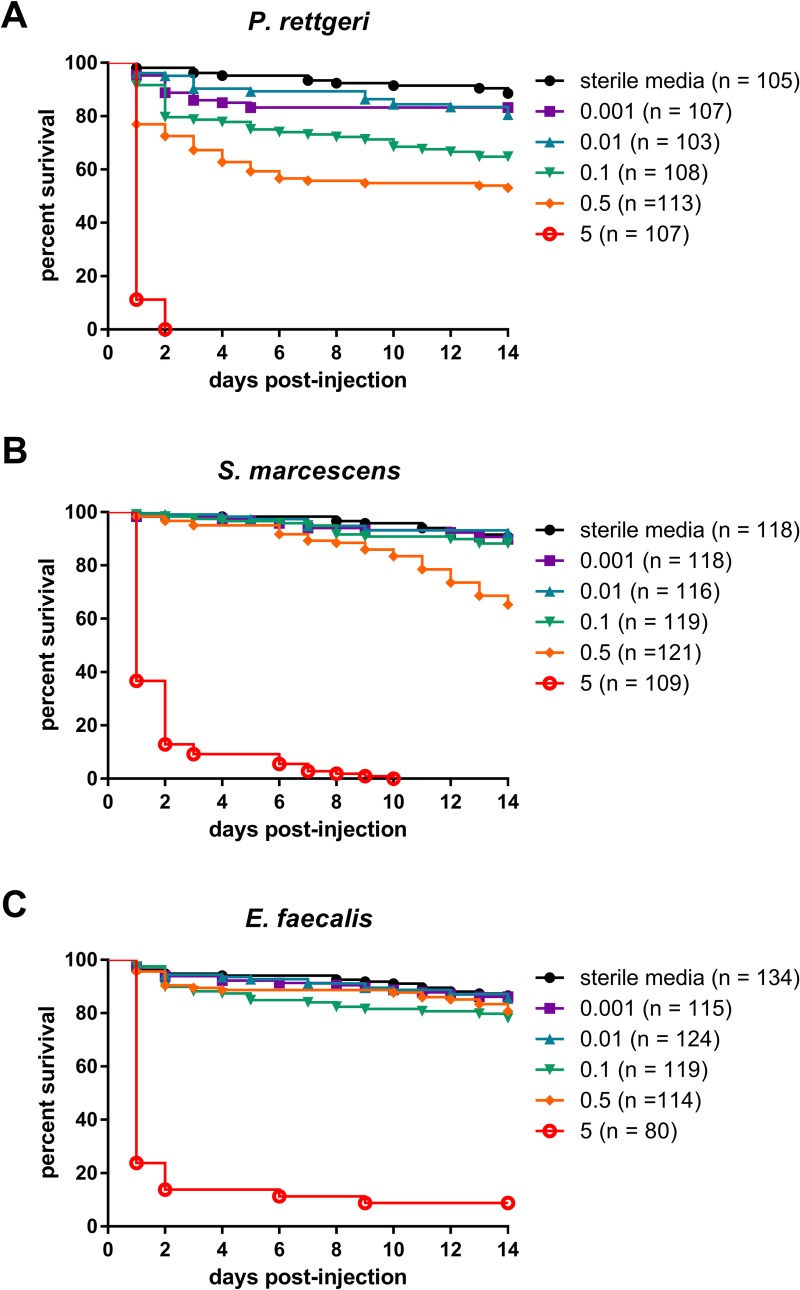
Survival of infection decreases as initial dose increases. Flies were injected with 23nL of bacterial suspension at the reported absorbance at 600nm and monitored for survival for 2 weeks. Results from experimental blocks were combined for graphical depiction.

As expected, mortality was dependent on infectious dose for all three species (p<0.01, Fig 1, Table 2), with the risk of death dramatically increasing with increasing dose. At the highest infectious dose of approximately 10^5^−10^6^ CFU per fly (A_600nm_ = 5), *P*. *rettgeri* infection caused 100% mortality within 2 days, *S*. *marcescens* infection caused 100% mortality within 10 days, and *E*. *faecalis* infection caused 93% mortality over two weeks (Fig 1). The lowest infectious dose, fewer than 100 bacteria per fly, caused less than 20% mortality after infection with each of the three bacterial species. *P*. *rettgeri* infection showed a strong dose-dependency for the risk of death even at the lower infectious doses whereas the other two bacteria elicited very little mortality except at the highest infection dose (Fig 1, Table 2).

**Table 2:** Impact of infectious dose on survival.

Bacterial species	Conditions included	Factor	χ2	Df	p-value
*P*. *rettgeri*	all doses	block	295.1	2	**<0.0001**
		log_10_ (infectious dose)	19.9	1	**<0.0001**
		block x log_10_ (infectious dose)	0.3	2	0.84
	0.001–0.5	block	26.1	2	**<0.0001**
		log_10_ (infectious dose)	10.8	1	**0.001**
		block x log_10_ (infectious dose)	1.1	2	0.57
*S*. *marcescens*	all doses	block	439.4	2	**<0.0001**
		log_10_ (infectious dose)	20.1	1	**<0.0001**
		block x log_10_ (infectious dose)	3.4	2	0.07
	0.001–0.5	block	237.3	2	**<0.0001**
		log_10_ (infectious dose)	9.8	1	**0.002**
		block x log_10_ (infectious dose)	1.8	2	0.40
*E*. *faecalis*	all doses	block	224.5	2	**<0.0001**
		log_10_ (infectious dose)	9.1	1	**0.003**
		block x log_10_ (infectious dose)	0.4	2	0.83
	0.001–0.5	block	0.7	2	0.72
		log_10_ (infectious dose)	2.7	1	0.10
		block x log_10_ (infectious dose)	9.1	2	**0.01**

Results from an ANOVA based on a Cox Proportional Hazards Mixed Effects model (Model A) used to assess the contribution of optical density of the bacterial suspension on survival. Optical density of the bacterial suspension was used to approximate infectious dose and log_10_ transformed prior to analysis.

**Fig 2:**
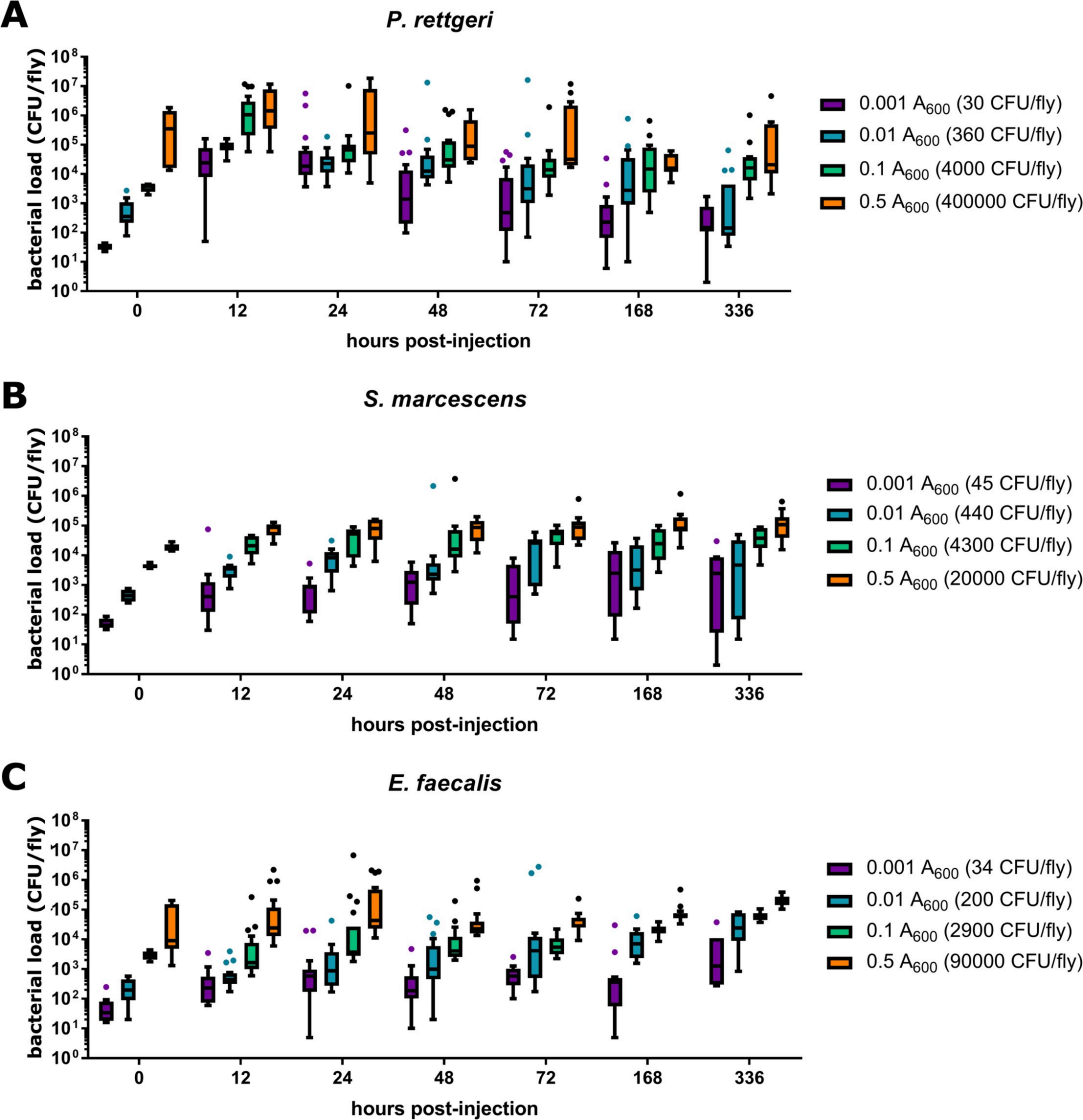
Bacterial load over time depends on infectious dose. Flies were injected with 23nL of bacterial suspension at the reported absorbance at 600nm and bacterial load assessed at the specified time-points post-injection. Median infectious dose for each absorbance is reported in the legend in parentheses. Results from experimental blocks were combined for graphical depiction.

We measured bacterial load at 12 hours, 24 hours, 48 hours, 72 hours, 7 days, and 14 days post-infection over a 500-fold range of inoculation doses, excluding the highest dose that caused complete and rapid mortality during the survival experiments. Infection with *P*. *rettgeri* was characterized by an initial increase in bacterial load after inoculation, but by three days post-infection, after most mortality had occurred, the load in survivors stabilized (Fig 2A). In contrast, there was little proliferation of *E*. *faecalis* or *S*. *marcescens* after the initial inoculation, which is consistent with the low mortality caused by these infections (Fig 2B and 2C). *E*. *faecalis* and *S*. *marcescens* chronic loads stabilized just above the level of the initial inoculum (Fig 2B and 2C). Infectious dose was a significant predictor of chronic load at both one and two weeks post-infection for all three bacterial species (p < 0.0001, Table 3), with increased inocula resulting in higher chronic loads sustained. This is consistent with previous work in the lab using these three species [2,5,9]. In two cases, we observed significant interaction terms between block and infectious dose (*S*. *marcescens* one-week post-infection, p = 0.03, and *E*. *faecalis* two-weeks post-infection, p = 0.0002; Table 3). In both cases, all blocks showed dose-dependence that was consistent in direction but the strength of the dependence was variable across blocks.

**Table 3:** Impact of infectious dose on persistent bacterial load.

Bacterial species	Time (week)	Factor	F value	Df	p-value
*P*. *rettgeri*	1	block	3.8	2	**0.03**
		log_10_ (infectious dose)	23.2	1	**<0.0001**
		block x log_10_ (infectious dose)	0.7	2	0.50
	2	block	2.9	2	0.07
		log_10_ (infectious dose)	63.6	1	**<0.0001**
		block x log_10_ (infectious dose)	1.0	2	0.38
*S*. *marcescens*	1	block	6.3	1	**0.02**
		log_10_ (infectious dose)	49.0	1	**<0.0001**
		block x log_10_ (infectious dose)	5.2	1	**0.03**
	2	block	3.1	1	0.09
		log_10_ (infectious dose)	34.1	1	**<0.0001**
		block x log_10_ (infectious dose)	0.4	1	0.52
*E*. *faecalis*	1	block	0.7	2	0.49
		log_10_ (infectious dose)	102.6	1	**<0.0001**
		block x log_10_ (infectious dose)	1.7	2	0.20
	2	block	3.8	1	0.06
		log_10_ (infectious dose)	64.0	1	**<0.0001**
		block x log_10_ (infectious dose)	17.2	1	**0.0002**

Results from an ANOVA (Model B, described in Methods) were used to assess the contribution of infectious dose on the persistent bacterial load at one and two weeks post-injection. Infectious dose was determined experimentally for each block and the average value was used in analysis. All bacterial loads were log_10_ transformed before analysis.

### Chronic infection results in sustained immune activation and resistance against secondary infection

The *Drosophila* immune system is stimulated by bacterial peptidoglycan, with DAP-type peptidoglycan (characteristic of Gram-negative bacteria and *Bacillus*) more strongly stimulating the Imd pathway and Lys-type peptidoglycan (characteristic of most Gram-positive bacteria) more strongly stimulating the Toll pathway [12,38]. Activation of each pathway leads to the expression of antimicrobial peptide genes, some of which are more elicited dominantly through either Imd or Toll and some of which are co-stimulated by both pathways [12,28]. Measuring the expression level of various antimicrobial peptide genes therefore provides an indication of the degree to which the different arms of the immune system are activated.

We assessed five antimicrobial peptide genes that have diverse immunological activity. First, we assessed *diptericin A*, which is strongly regulated by the Imd pathway [12] and is the primary determinant of susceptibility to *P*. *rettgeri* infection [39,40]. We additionally assayed expression of two other antimicrobial peptide genes (*attacin A* and *metchnikowin*) that can be induced by both Toll and Imd signaling and two antimicrobial peptide genes (*defensin* and *drosomycin*) that are most strongly regulated by the Toll pathway [12]. These peptides have antimicrobial effects against different types of pathogens [39,41–44].

**Fig 3:**
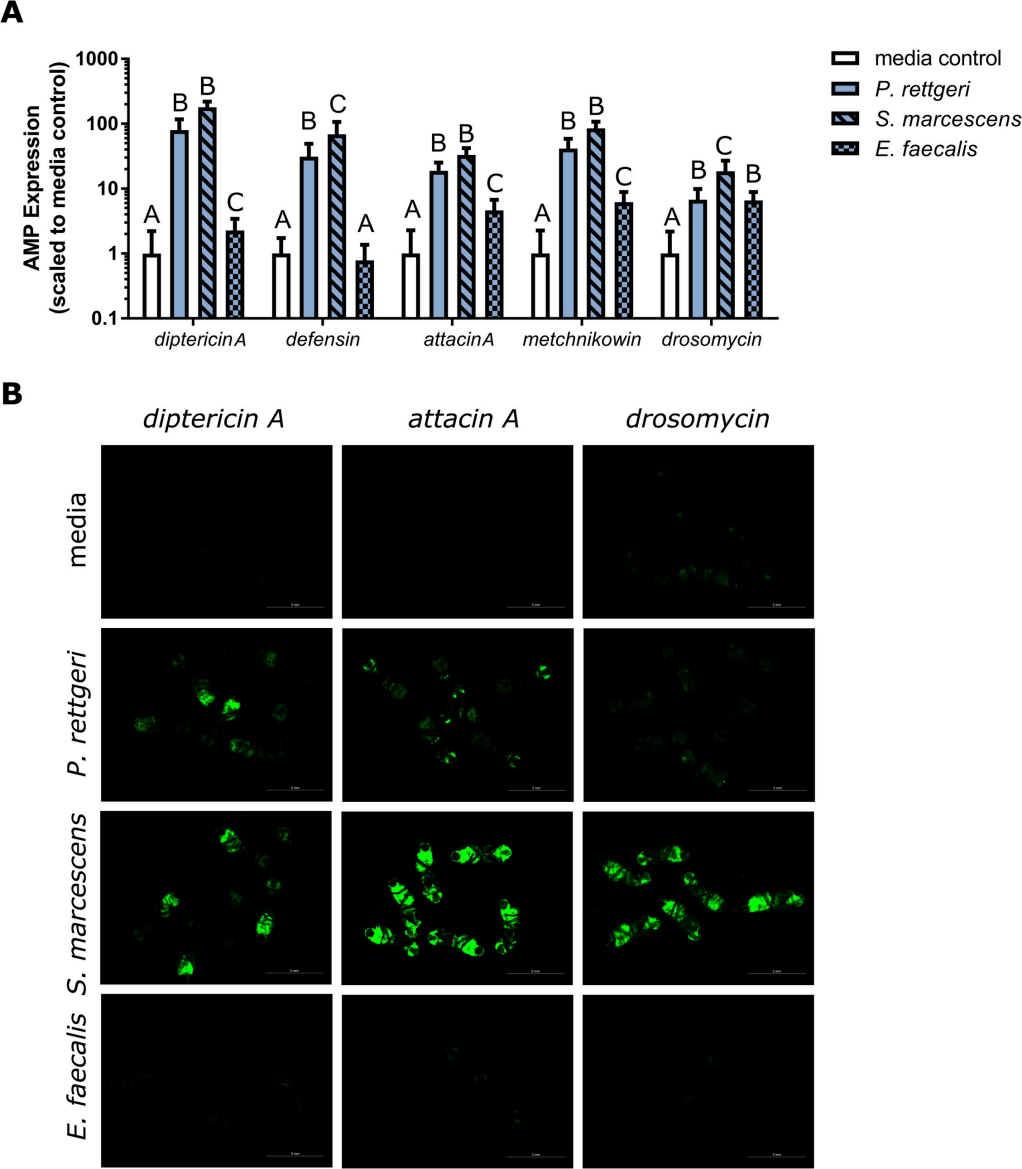
Antimicrobial peptide gene expression one week post-infection. Flies were injected in the abdomen with 23nL of bacterial suspension (A_600nm_ = 0.1; approximately 2000–6000 CFU) and antimicrobial peptide transcription was assessed at one week post-injection by qPCR (A) and through use of AMP-promoter>GFP constructs (B). (A) Relative expression was calculated using the Pfaffl method and was scaled to media-injected controls for graphical representation. Significantly different expression values across treatment within each antimicrobial peptide transcript were determined by Tukey’s multiple comparison test and are indicated by the capital letters, where treatments that share a letter are not significant with *p* > 0.01. More detailed results of the statistical analysis are in S1 and S2 Tables. (B) All panels contain 6 flies except the *E*. *faecalis*-*drosomycin* panel, which contains only 2 flies due to high mortality, and images for each reporter construct (*diptericin A*, *attacin A* and *drosomycin*) were taken with the same exposure for each infection condition. The scale bar indicates 2mm, and corresponding brightfield images are shown in S1 Fig.

We hypothesized that chronic infection would result in sustained expression of antimicrobial peptides. To test this, we infected flies with *P*. *rettgeri*, *S*. *marcescens*, and *E*. *faecalis* at a dose that is expected to yield a chronic burden of approximately 10^4^ bacteria per fly and measured antimicrobial peptide gene expression by qPCR and GFP fluorescence from reporter constructs. Consistent with our hypothesis, we found that chronic infection with all three infections resulted in significantly upregulated expression of *diptericin A*, *attacin A*, *metchnikowin*, and *drosomycin* at one-week post-infection, although infection with the Gram-positive pathogen *E*. *faecalis* stimulated lower levels of antimicrobial peptide gene expression than infections with *P*. *rettgeri* and *S*. *marcescens* (Fig 3A). In contrast, *defensin* was only significantly upregulated by the Gram-negative pathogens, *S*. *marcescens* and *P*. *rettgeri* (Fig 3A). This can also be shown visually with reporter lines that express green fluorescent protein (GFP) under the control of antimicrobial peptide promoters. These reporter lines generally exhibited comparable mortality to Canton S flies of 10–20%, except for *E*. *faecalis* infection of the drosomycin reporter line, which unexpectedly resulted in 80% mortality. GFP fluorescence driven by *diptericin A*, *attacin A*, and *drosomycin* promoters was strong at one week post-infection with *S*. *marcescens* (Fig 3B). GFP expression from the Imd-regulated *diptericin A* and *attacin A* promoters was moderate at one week post-infection with *P*. *rettgeri* but was much weaker from the Toll-regulated *drosomycin* promoter (Fig 3B), despite the continued significant upregulation of the native *drosomycin* gene as measured by qPCR (Fig 3A). In contrast, reporter lines infected with *E*. *faecalis* showed negligible GFP expression above controls (Fig 3B). These results demonstrate sustained immune activation from chronic infection with all three chronic infections, although a much weaker elicitation from chronic *E*. *faecalis* infection.

**Fig 4:**
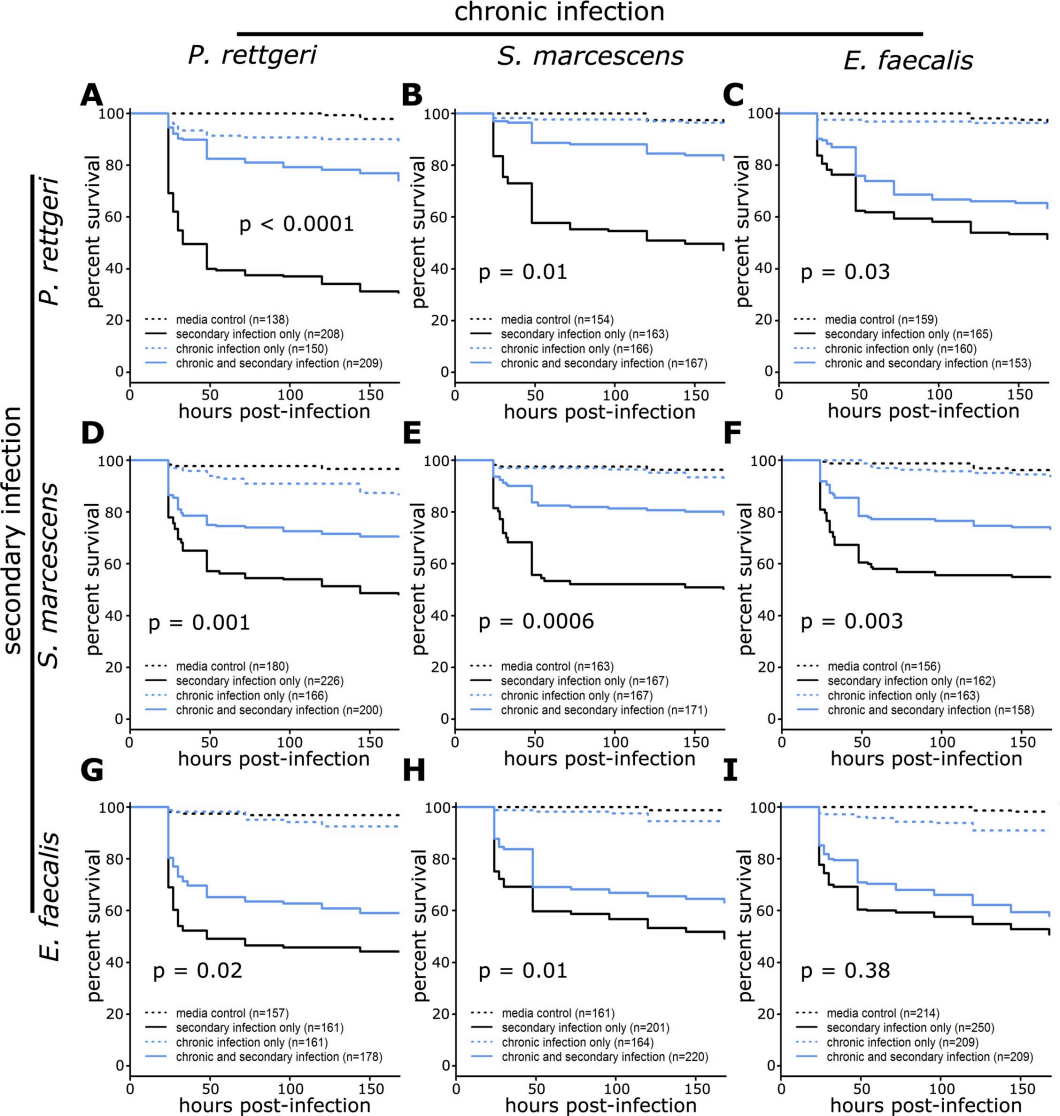
Chronically infected flies better survive secondary bacterial infections. Flies were injected with 23nL of bacterial suspension (A_600nm_ = 0.1) or sterile media and maintained at 25°C for one week to allow chronic infection to develop. The flies were then given a secondary injection by injecting 23nL of bacterial suspension (A_600nm_ = 0.1) or sterile media into the thorax. Host survival was monitored for one week. Chronic infection for each panel is as follows: *P*. *rettgeri* (A, D, G), *S*. *marcescens* (B, E, H), and *E*. *faecalis* (C, F, I). Secondary infections for each panel is as follows: *P*. *rettgeri* (A, B, C), *S*. *marcesens* (D, E, F), and *E*. *faecalis* (G, H, I). Results from experimental blocks were combined for graphical depiction. The statistical significance of the protective effect is overlaid on each panel with the complete statistics provided in Table 4.

We next hypothesized that sustained immune activation elicited by chronic infection might result in protection against secondary bacterial infection. To test this, we chronically infected flies with each of the three pathogens and subsequently subjected them to a more intense challenge with either the same pathogen or one of the other two. Expected lethality from the secondary infection was increased by delivering the inoculum into the thorax, which results in increased bacterial proliferation and higher levels of mortality [2]. Overall, chronic infection was protective against mortality from secondary infection, with eight out of nine combinations of chronic and secondary infection showing significant protection (Fig 4; Table 4). More specifically, chronic infection with *P*. *rettgeri* provided significant protection against all three secondary infections (*P*. *rettgeri*: p<0.0001; *S*. *marcescens*: p = 0.001, *E*. *faecalis*: p = 0.02). Chronic infection with *S*. *marcescens* gave significant protection against all three secondary infections (*P*. *rettgeri*: p = 0.01; *S*. *marcescens*: p = 0.0006, *E*. *faecalis*: p = 0.01). Chronic infection with *E*. *faecalis* also gave significant protection against *P*. *rettgeri* (p = 0.03) and *S*. *marcescens* (p = 0.003). Surprisingly, *E*. *faecalis* chronic infection did not give significant protection against secondary infection with *E*. *faecalis* (p = 0.38) although the data trend in the direction of protection. For the two combinations that showed a significant block by infection interaction (*P*. *rettgeri*-*P*. *rettgeri*: p = 0.03, *S*. *marcescens*-*P*. *rettgeri*: p = 0.003), every block individually showed that flies carrying chronic infection and secondary infection survived better than flies only carrying the secondary infection, although the magnitude of this difference varied among individual blocks.

**Table 4:** Effect of chronic infection on survival to a secondary acute infection.

Chronic Infection	Secondary Infection	Factor	χ^2^	Df	p-value
*P*. *rettgeri*	*P*. *rettgeri*	block	112.6	3	**<0.0001**
		chronic infection	16.6	1	**<0.0001**
		block x chronic infection	9.1	3	**0.03**
	*S*. *marcescens*	block	24.7	3	**<0.0001**
		chronic infection	10.6	1	**0.001**
		block x chronic infection	2.5	3	0.48
	*E*. *faecalis*	block	9.9	2	**0.007**
		chronic infection	5.1	1	**0.02**
		block x chronic infection	2.9	2	0.23
*S*. *marcescens*	*P*. *rettgeri*	block	51.6	2	**<0.0001**
		chronic infection	6.5	1	**0.01**
		block x chronic infection	11.9	2	**0.003**
	*S*. *marcescens*	block	35.7	2	**<0.0001**
		chronic infection	11.6	1	**0.0006**
		block x chronic infection	0.5	2	0.77
	*E*. *faecalis*	block	14.4	2	**0.0007**
		chronic infection	6.5	1	**0.01**
		block x chronic infection	0.8	2	0.67
*E*. *faecalis*	*P*. *rettgeri*	block	3.9	2	0.14
		chronic infection	4.8	1	**0.03**
		block x chronic infection	1.9	2	0.39
	*S*. *marcescens*	block	34.6	2	**<0.0001**
		chronic infection	9.4	1	**0.003**
		block x chronic infection	0.4	2	0.84
	*E*. *faecalis*	block	29.3	3	**<0.0001**
		chronic infection	0.8	1	0.38
		block x chronic infection	2.4	3	0.49

The impact of chronic infection on survival to secondary infection was assessed using a Cox Proportional Hazards Mixed Effects model (Model D, see Materials and Methods) followed by an ANOVA.

### Chronic infection with *Providencia rettgeri* causes decreased starvation resistance

**Fig 5:**
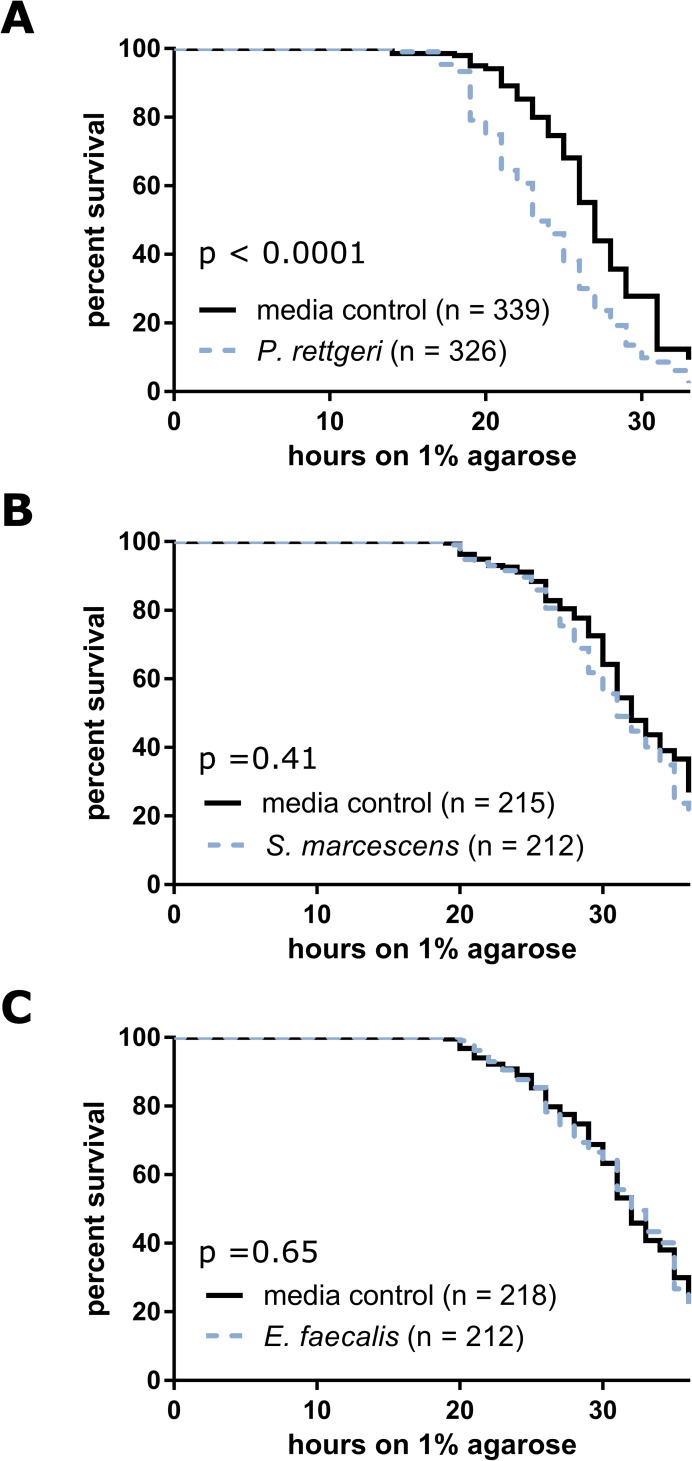
Flies chronically infected with *P*. *rettgeri* are more susceptible to starvation. Survival of chronically infected flies during starvation stress was compared to uninfected controls by flipping the flies to 1% non-nutritive agarose and monitoring survival for 35 hours. Results from experimental blocks were combined for graphical depiction. The statistical significance of chronic infection’s impact on starvation resistance is overlaid on each panel with the complete statistics provided in Table 5.

It seems plausible that bearing a substantial chronic infection could be energetically costly, in part due to the costs of sustained immune activation. Time to starvation in the absence of nutrients provides a reliable indication of overall energy state [45]. To assess whether chronic infection puts an energetic strain on the host, we subjected chronically infected flies to intense starvation stress by flipping them to non-nutritious agarose for 35 hours. We monitored the flies’ survival at one hour intervals. Flies infected with *P*. *rettgeri* died significantly faster than controls (p<0.0001, Fig 5A, Table 5). While flies with chronic *S*. *marcescens* infections appeared to starve marginally more quickly than controls, the effect was not significant (p = 0.41, Fig 5B, Table 5). Due to the significant interaction between block and *S*. *marcescens* infection (p = 0.02, Table 5), we examined each block individually and found that increased sensitivity to starvation occurred in only a single block and that the other two blocks showed no effect of chronic infection. Chronic infection with *E*. *faecalis* also did not cause any difference in starvation sensitivity (p = 0.65, Fig 5C, Table 5).

**Table 5:** Effect of chronic infection of starvation resistance.

Chronic Infection	Factor	χ^2^	Df	p-value
*P*. *rettgeri*	block	215.1	5	**<0.0001**
	chronic infection	18.8	1	**<0.0001**
	block x chronic infection	4.9	5	0.43
*S*. *marcescens*	block	96.8	2	**<0.0001**
	chronic infection	0.7	1	0.41
	block x chronic infection	8.4	2	**0.02**
*E*. *faecalis*	block	125.2	2	**<0.0001**
	chronic infection	0.2	1	0.65
	block x chronic infection	3.1	2	0.21

The contribution of chronic infection to survival during starvation was assessed using a Cox Proportional Hazards Mixed Effects model (Model D, see Materials and Methods) followed by an ANOVA.

## Discussion

In this study, we demonstrate that diverse bacteria are able to establish chronic infections in the *D*. *melanogaster* host (Figs 1 and 2), and that the number of chronically persistent bacteria is predicted by the initial inoculation dose (Table 3). Chronic infection results in chronically upregulated immune reactions (Fig 3), and in turn is partially protective against secondary infection (Fig 4, Table 4). This protection is nonspecific and could potentially be explained by sustained production of antimicrobial peptides. We found that chronic infection with *P*. *rettgeri* caused host flies to become sensitive to starvation (Fig 5, Table 5), suggesting an energetic cost of bearing that infection. This work lays an initial foundation for studies of the causes and consequences of chronic infection in the experimentally tractable *D*. *melanogaster* host.

Diverse bacterial species are able to establish chronic infection in *D*. *melanogaster*. These include *Providencia rettgeri* [1,2,5, present study], *Serratia marcescens* [9, present study], *Escherichia coli* [5,9] *Pectobacterium* (formerly *Erwinia*) *carotovora* [5,9], *Enterococcus faecalis* [2,5,9, present study], *Lactococcus lactis* [4], and several other species of *Providencia* (unpublished observations). All of these are opportunistic infectors of *Drosophila* and other organisms, and they are in some cases very distantly related to each other. It seems unlikely, then, that they share a specialized adaptation for infection of an insect host. We suspect it is much more likely that the chronic infection emerges through a common property of bacterial physiology, possibly adaptive for withstanding harsh abiotic environmental conditions. It is then incidentally triggered in a subset of infected *Drosophila* leading to a relatively asymptomatic infection that the host is able to tolerate.

We observed here that the chronic bacterial load was correlated with the initial infection dose for *P*. *rettgeri*, *S*. *marcescens* and *E*. *faecalis* (Fig 2), as was previously observed for *P*. *rettgeri* [5]. Duneau et al. [5] proposed a model to describe bacterial infection in *D*. *melanogaster*. In this model, the bacteria grow logarithmically over the first few hours of infection, during a lag time in which the host immune response is becoming activated (termed *t*^c^ in that paper). Duneau et al. hypothesized a critical threshold for bacterial density, proposing that if the bacteria reach this threshold prior to sufficient activation of the host immune response, the bacteria would continue to proliferate and would kill the host. However, if the bacteria fail to reach this threshold density, it was hypothesized that the infection would freeze at whatever density had been reached at *t*^c^ and become chronic. Our data are consistent with this model. Assuming a constant rate of net bacterial proliferation, higher inoculation doses would be expected to yield a higher probability of death because the infection begins closer to the critical threshold, and surviving flies would be expected to bear higher pathogen burdens during the chronic phase. We observe both of these phenomena in our results, lending support for this theoretical model.

We found that chronic infection with all three bacteria stimulates chronic upregulation of *D*. *melanogaster* genes encoding antimicrobial peptides (Fig 3), although this immune activation is clearly not sufficient to eliminate the infections (Fig 2). Notably, both of the Gram-negative bacteria–*P*. *rettgeri* and *S*. *marcescens*—stimulate sustained induction of antimicrobial peptide genes that are regulated by both the Toll and Imd pathways, despite the canonical expectation that Gram-negative bacteria should predominantly stimulate Imd signaling [12,17,46]. Although chronic infection with the Gram-positive *E*. *faecalis* stimulates increased expression of four of the five antimicrobial peptide genes tested (Fig 3A), it does not show significant upregulation of *defensin*, a gene that encodes a peptide with potent antibacterial properties against Gram-positive bacteria that can be elicited by stimulation of the Toll pathway [12,17,46]. This adds to an increasing body of evidence that the Toll and Imd pathways do not dichotomously and simplistically discriminate between Gram-positive and Gram-negative infections [9,47–49]. The differences among the three bacteria in the degree of chronic immune activation is not due to differences in the chronic bacterial load, as all three bacteria persist at similar levels (Fig 2), but *S*. *marcescens* stimulates a much stronger sustained immune reaction and *E*. *faecalis* stimulates a much weaker one (Fig 3). Earlier work looking at the correlation between bacterial load and antimicrobial peptide gene expression in *D*. *melanogaster*, *Tribolium castaneum*, and *Bombus terrestris* focused on a single bacterial species within each host: *Listeria monocytogenes*, *Bacillus thuringiensis*, and *Escherichia coli*, respectively [19–21]. However, our findings show that strength of immune stimulation is highly dependent on bacterial species and suggest that the relationship between bacterial load and immune stimulation may vary across host-bacteria pairings.

We additionally found that chronic infection is generically protective against secondary infection (Fig 4, Table 4) with no evidence of specificity in that protection. The two bacteria that stimulated the most elevated expression of antimicrobial peptide genes relative to control during the chronic phase of infection, *P*. *rettgeri* and *S*. *marcescens* (Fig 3), provided the most robust protection against secondary infection. Our finding of elevated antimicrobial peptides gene expression suggests that this protection is likely due to increased killing of the secondary infection, but other factors that could influence protection during secondary infection include competition among the chronically and secondarily infecting bacteria for resources and/or increased tolerance of infection by the host. It is also possible that the ten to twenty percent mortality during the first week of the initial infection (Fig 1) selects for the subset of flies that are more robust to secondary infection. However, this is unlikely to account for the entire protective effect we observe. Our results show that chronic infection can increase survival of secondary infection by thirty percent or greater compared to flies without a chronic infection (Fig 4A, 4B & 4D). This suggests that if selection plays a role, it works in combination with other factors.

Our findings support the idea that immune stimulation by one systemic infection may be generally protective against subsequent systemic infections in insects. Work in the caterpillar *Manduca sexta* demonstrated that infection with non-pathogenic *Escherichia coli* provided protection against later infection with the pathogenic *Photorhabdus luminescens* [50]. This is in contrast to other studies in both *D*. *melanogaster* and the red flour beetle (*Tribolium casteum*), which have shown specificity in the protective effect of prior exposure to bacteria [24,51]. This may result from differences in experimental methodology as both of those experiments used heat-killed bacteria to test the specificity of their responses. It is possible that live bacterial infection is be required to elicit generalized protection against future challenges. In the buff-tailed bumblebee (*Bombus terrestris)* there is also evidence for cross-protection, although protection was stronger for prior sub-lethal infection with the same pathogen than with another bacterial species [52]. In that experiment, the initial infections were cleared and no longer present at the time of secondary infection, which could have reduced the intensity of any sustained immune stimulation relative to what we saw in our experiment, where the initial infection was sustained throughout the secondary infection as well.

Sustaining a long-term infection seems potentially costly because of the energetic demands of sustaining the bacteria, the demands of chronically sustained immune reactions, or both. We used resistance to starvation as an indirect measure of the energetic state of the host [45]. Surprisingly, only chronic infections with *P*. *rettgeri* put enough strain on the host to make them significantly more susceptible to starvation (Fig 5). Chronic *S*. *marcescens* and *E*. *faecalis* infections did not reduce resistance to starvation. Interestingly, chronic infection with *P*. *rettgeri* did not significantly impact female fecundity [1] or lifespan [2] in previous studies. Thus, chronic infection appears to have only limited energetic consequence, despite appreciable bacterial loads (Fig 2) and chronic upregulation of the immune response (Fig 3). This finding is somewhat surprising because sustained infections by other bacteria have been shown to deplete energy stores [53–55] and there is evidence for a trade-off between immune response and energy storage [1,56]. However, the bacteria causing the most dramatic impact on energy storage in previous studies established intracellular infections that may be more physiologically disruptive or energetically demanding than our current extracellular chronic infections. Additionally, perhaps the level of antimicrobial peptide gene expression we observed does not place a large demand on host energetic resources. Nevertheless, there is a modest cost to sustaining chronic infection with *P*. *rettgeri* even under laboratory conditions, and costs in the field may be even more severe.

Overall, this work demonstrates that chronic infection of *Drosophila melanogaster* is a general phenomenon that can be established by multiple distantly related bacteria, and with varied physiological impact on the host. The chronic infection seems to offer nonspecific protection against secondary bacterial infection, although any benefit from the primary infection needs to be weighed against the risk of death from that initial infection. Chronic infection upregulates immune responses, with the intensity of that upregulation depending more on the identity of the infecting bacterium than it does on the bacterial titer. Chronic infection can also carry energetic cost to the host, although this too varies among pathogens and is not strictly predicted by bacterial titer or magnitude of immune induction. Determining the mechanistic origins of these effects on the host, as well as how the bacteria are able to establish non-lethal chronic infection, should be fruitful areas for future study.

## Supporting information

S1 Fig Brightfield images for chronically infected GFP reporter flies. Flies were injected in the abdomen with 23nL of bacterial suspension (A_600nm_ = 0.1) and antimicrobial peptide transcription assessed at one week post-injection by through use of promoter-GFP constructs (Fig 3B). All panels contain 6 flies except the *E*. *faecalis-drosomycin* panel which contains 2 flies due to higher mortality, and each reporter construct (*diptericin A*, *attacin A* and *drosomycin*) was exposed for the same amount of time across conditions. Scale bar indicates 2mm.
(TIF)

S1 File Data for Publication. This compressed folder contains all of the data for the manuscript and the data files are titled with their corresponding figure panel.
(ZIP)

S1 Table Results from ANOVA analysis for antimicrobial peptide gene expression. The contribution of infection seven days post-infection to antimicrobial peptide gene expression was assessed by ANOVA (model C).
(DOCX)

S2 Table Results from Tukey HSD post-hoc analysis on antimicrobial peptide gene expression. To determine which infections had a significant impact on antimicrobial peptide gene expression, a post-hoc Tukey test was performed on our linear model to do pair-wise comparisons between each infection conditions.
(DOCX)
