# Supplementary figures and images for: Consequences of chronic bacterial infection in Drosophila melanogaster
Source: PLoS One. 2019 Oct 24;14(10):e0224440. doi: 10.1371/journal.pone.0224440 (PMC6812774; doi:10.1371/journal.pone.0224440)

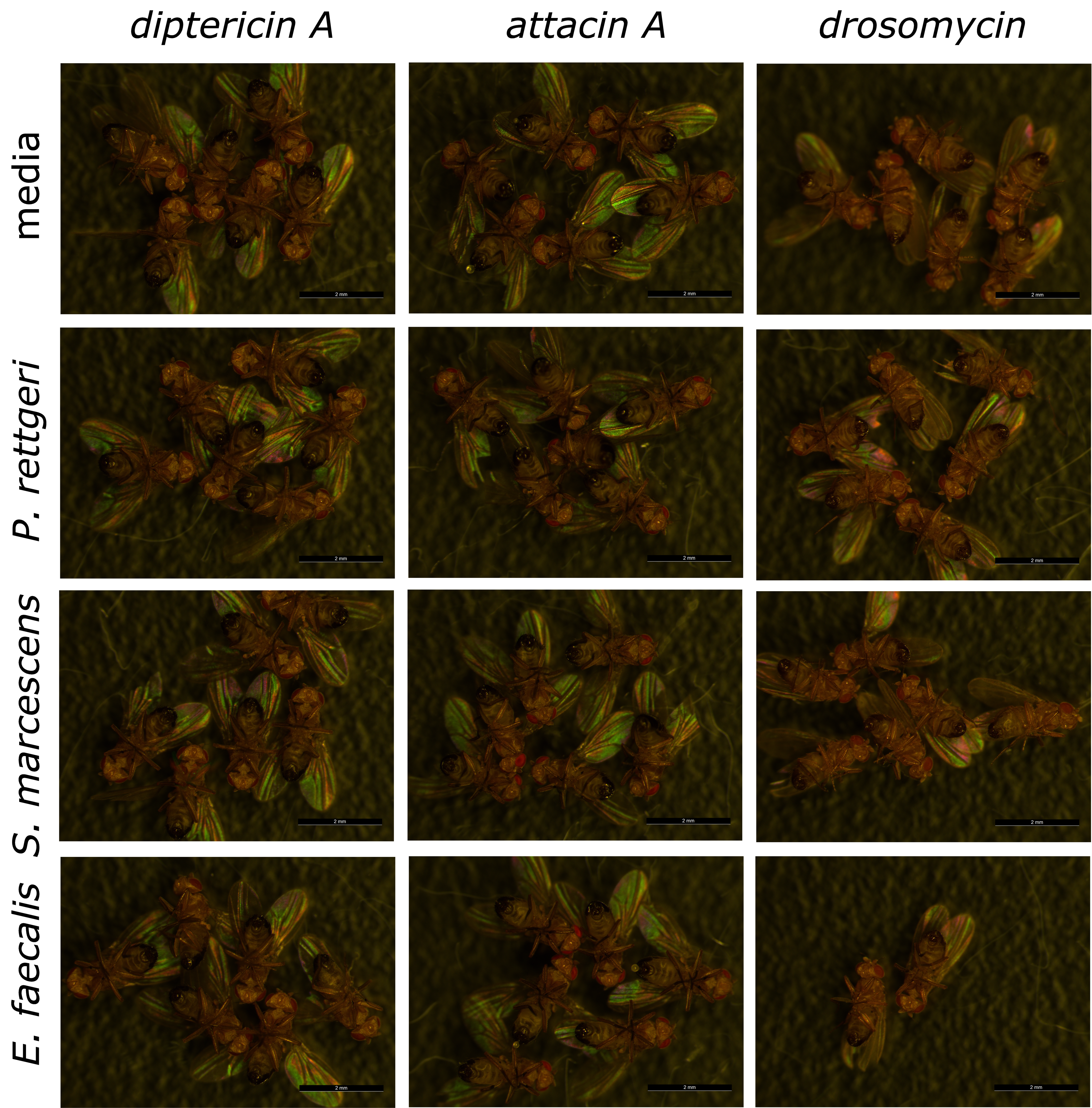

Supplement: S1 Fig — Flies were injected in the abdomen with 23nL of bacterial suspension (A600nm = 0.1) and antimicrobial peptide transcription assessed at one week post-injection by through use of promoter-GFP constructs (Fig 3B). All panels contain 6 flies except the E. faecalis-drosomycin panel which contains 2 flies due to higher mortality, and each reporter construct (diptericin A, attacin A and drosomycin) was exposed for the same amount of time across conditions. Scale bar indicates 2mm. (TIF) [file pone.0224440.s001.tif]
