# Supplementary material for: Consequences of chronic bacterial infection in Drosophila melanogaster
Source: PLoS One. 2019 Oct 24;14(10):e0224440. doi: 10.1371/journal.pone.0224440 (PMC6812774; doi:10.1371/journal.pone.0224440)
Supplement: S1 Table — The contribution of infection seven days post-infection to antimicrobial peptide gene expression was assessed by ANOVA (model C). (DOCX) [file pone.0224440.s003.docx]

**S1 Table. Results from ANOVA analysis for antimicrobial peptide gene expression.**

| Antimicrobial peptide gene | Factor | F-value | Df | p-value |
| --- | --- | --- | --- | --- |
| *diptericin A* | block | 9.1 | 2 | **0.001** |
|  | injection | 181.0 | 3 | **<0.0001** |
|  | block x injection | 3.6 | 6 | 0.01 |
| *defensin* | block | 12.1 | 2 | **<0.0001** |
|  | injection | 211.4 | 3 | **<0.0001** |
|  | block x injection | 4.1 | 6 | **0.006** |
| *attacin A* | block | 8.2 | 2 | **0.001** |
|  | injection | 96.0 | 3 | **<0.0001** |
|  | block x injection | 4.6 | 6 | **0.003** |
| *metchnikowin* | block | 9.1 | 2 | **0.001** |
|  | injection | 116.3 | 3 | **<0.0001** |
|  | block x injection | 4.1 | 6 | **0.006** |
| *drosomycin* | replicate | 8.5 | 2 | **0.001** |
|  | injection | 61.1 | 3 | **<0.0001** |
|  | replicate x injection | 1.6 | 6 | 0.18 |

The contribution of infection seven days post-infection to antimicrobial peptide gene expression was assessed by ANOVA (model C).
