# Supplementary material for: Consequences of chronic bacterial infection in Drosophila melanogaster
Source: PLoS One. 2019 Oct 24;14(10):e0224440. doi: 10.1371/journal.pone.0224440 (PMC6812774; doi:10.1371/journal.pone.0224440)
Supplement: S2 Table — To determine which infections had a significant impact on antimicrobial peptide gene expression, a post-hoc Tukey test was performed on our linear model to do pair-wise comparisons between each infection conditions. (DOCX) [file pone.0224440.s004.docx]

**S2 Table. Results from Tukey HSD post-hoc analysis on antimicrobial peptide gene expression.**

| Antimicrobial peptide gene | comparison (injection) | p-value |
| --- | --- | --- |
| *diptericin A* | Sterile PBS – *P. rettgeri* | **<0.0001** |
|  | Sterile PBS – *S. marcescens* | **<0.0001** |
|  | Sterile PBS – *E. faecalis* | **<0.0001** |
|  | *P. rettgeri* – *S. marcescens* | 0.03 |
|  | *P. rettgeri* – *E. faecalis* | **<0.0001** |
|  | *S. marcescens* – *E. faecalis* | **<0.0001** |
| *defensin* | Sterile PBS – *P. rettgeri* | **<0.0001** |
|  | Sterile PBS – *S. marcescens* | **<0.0001** |
|  | Sterile PBS – *E. faecalis* | 0.76 |
|  | *P. rettgeri* – *S. marcescens* | **0.004** |
|  | *P. rettgeri* – *E. faecalis* | **<0.0001** |
|  | *S. marcescens* – *E. faecalis* | **<0.0001** |
| *attacin A* | Sterile PBS – *P. rettgeri* | **<0.0001** |
|  | Sterile PBS – *S. marcescens* | **<0.0001** |
|  | Sterile PBS – *E. faecalis* | **<0.0001** |
|  | *P. rettgeri* – *S. marcescens* | 0.23 |
|  | *P. rettgeri* – *E. faecalis* | **0.0003** |
|  | *S. marcescens* – *E. faecalis* | **<0.0001** |
| *metchnikowin* | Sterile PBS – *P. rettgeri* | **<0.0001** |
|  | Sterile PBS – *S. marcescens* | **<0.0001** |
|  | Sterile PBS – *E. faecalis* | **<0.0001** |
|  | *P. rettgeri* – *S. marcescens* | 0.11 |
|  | *P. rettgeri* – *E. faecalis* | **0.0003** |
|  | *S. marcescens* – *E. faecalis* | **<0.0001** |
| *drosomycin* | Sterile PBS – *P. rettgeri* | **<0.0001** |
|  | Sterile PBS – *S. marcescens* | **<0.0001** |
|  | Sterile PBS – *E. faecalis* | **<0.0001** |
|  | *P. rettgeri* – *S. marcescens* | **0.003** |
|  | *P. rettgeri* – *E. faecalis* | 0.98 |
|  | *S. marcescens* – *E. faecalis* | **0.005** |

To determine which infections had a significant impact on antimicrobial peptide gene expression, a post-hoc Tukey test was performed on our linear model to do pair-wise comparisons between each infection conditions.
